# Dysregulation of the EphrinB2−EphB4 ratio in pediatric cerebral arteriovenous malformations is associated with endothelial cell dysfunction in vitro and functions as a novel noninvasive biomarker in patients

**DOI:** 10.1038/s12276-020-0414-0

**Published:** 2020-04-14

**Authors:** Katie Pricola Fehnel, David L. Penn, Micah Duggins-Warf, Maxwell Gruber, Steven Pineda, Julie Sesen, Alexander Moses-Gardner, Nishali Shah, Jessica Driscoll, David Zurakowski, Darren B. Orbach, Edward R. Smith

**Affiliations:** 1 Vascular Biology Program, Boston Children’s Hospital, Boston, MA USA; 2 Department of Neurosurgery, Boston Children’s Hospital, Boston, MA USA; 3 Department of Surgery, Boston Children’s Hospital, Boston, MA USA

**Keywords:** Neuro-vascular interactions, Diagnostic markers

## Abstract

We investigated (1) EphrinB2 and EphB4 receptor expression in cerebral AVMs, (2) the impact of an altered EphrinB2:EphB4 ratio on brain endothelial cell function and (3) potential translational applications of these data. The following parameters were compared between AVM endothelial cells (AVMECs) and human brain microvascular endothelial cells (HBMVECs): quantified EphrinB2 and EphB4 expression, angiogenic potential, and responses to manipulation of the EphrinB2:EphB4 ratio via pharmacologic stimulation/inhibition. To investigate the clinical relevance of these in vitro data, Ephrin expression was assessed in AVM tissue (by immunohistochemistry) and urine (by ELISA) from pediatric patients with AVM (*n* = 30), other cerebrovascular disease (*n* = 14) and control patients (*n* = 29), and the data were subjected to univariate and multivariate statistical analyses. Compared to HBMVECs, AVMECs demonstrated increased invasion (*p* = 0.04) and migration (*p* = 0.08), impaired tube formation (*p* = 0.06) and increased EphrinB2:EphB4 ratios. Altering the EphrinB2:EphB4 ratio (by increasing EphrinB2 or blocking EphB4) in HBMVECs increased invasion (*p* = 0.03 and *p* < 0.05, respectively). EphrinB2 expression was increased in AVM tissue, which correlated with increased urinary EphrinB2 levels in AVM patients. Using the optimal urinary cutoff value (EphrinB2 > 25.7 pg/μg), AVMs were detected with high accuracy (80% vs. controls) and were distinguished from other cerebrovascular disease (75% accuracy). Post-treatment urinary EphrinB2 levels normalized in an index patient. In summary, AVMECs have an EphrinB2:EphB4 ratio that is increased compared to that of normal HBMVECs. Changing this ratio in HBMVECs induces AVMEC-like behavior. EphrinB2 is clinically relevant, and its levels are increased in AVM tissue and patient urine. This work suggests that dysregulation of the EphrinB2:EphB4 signaling cascade and increases in EphrinB2 may play a role in AVM development, with potential utility as a diagnostic and therapeutic target.

## Blood vessels: untangling the causes of brain vessel malformations

Tangled blood vessel growths in the brain, known as arteriovenous malformations (AVMs), can be identified with a urine test, and the test protein may also help in treatment. AVMs often have no symptoms and can go undiagnosed, but when they rupture they can cause deadly brain hemorrhage. Better diagnostic tools and nonsurgical treatments are needed. Katie Fehnel and Edward Smith at Boston Children’s Hospital, USA, and co-workers identified an imbalance in a pair of signal/receptor proteins called ephrins in AVMs. Disturbing the balance of ephrin levels in blood vessel-forming cells disrupted growth, causing disorganized vessel formation with too many sprouts and insufficient junctions. Testing ephrin levels in patients’ urine reliably identified AVMs. These results offer a rapid and noninvasive new diagnostic tool and may help find new treatments for this mostly invisible and potentially fatal condition.

## Introduction

Cerebral arteriovenous malformations (AVMs) are vascular anomalies with an average hemorrhage risk of 2–4% per year cumulative over a patient’s lifetime, and a 25% fatality risk with each bleed^1^. The morbidity and mortality of AVMs is compounded by limitations in effective and cost-efficient detection methods and even further by existing high-risk interventional treatments without therapeutic options for the some of the highest risk lesions. There is a need for better diagnostic techniques and novel therapeutic targets to reduce risk and improve patient outcomes, particularly in the pediatric population.

We have identified axon guidance factors (AGFs), specifically EphrinB2 and EphB4, as promising biomarkers of pediatric AVM. Ephrins are cell surface proteins that are readily quantifiable and potentially amenable to manipulation; they play a critical role in normal cerebrovascular development, proliferation, migration, and adhesion, through complex bidirectional signaling (forward through EphB4 receptor, reverse through EphrinB2 ligand). Arteriovenous cell fate determination occurs through canonical EphB4 signaling, and later angiogenesis, including vessel sprouting and remodeling, occurs through EphrinB2^2–4^. Inhibition of EphrinB2 signaling can suppress endothelial cell migration and tube formation in vitro, and it can lead to aberrations in the actin cytoskeleton^4^. In addition to high affinity binding between the extracellular EphB4 binding domain to EphrinB2, there are additional lower affinity residues on the EphB4 receptors that allow for homodimerization as well as tetramer aggregation^5^. Aggregate binding can alter the strength of signaling with downstream effects influenced by the ratio of ligand to receptor. These data suggest that a balance of ligand to receptor is critical to influencing downstream vascular fate.

Ephrins have been implicated in AVM-like pathology in animal models^6–8^. Transgenic mice with loss of either Ephb4 or EphrinB2 are embryonically lethal (at E9.5), and they exhibit an AVM-like pathology with shunting of arterial blood via fusion of the dorsal aorta with the common cardinal vein^9–12^. Vascular endothelial-specific overexpression of EphrinB2 in mice results in intracranial hemorrhage^13^. Recent literature further suggests that AGFs play a critical role in pathological vascular development in the brain. EphrinB2 is expressed in hemorrhagic AVM endothelium^14^, and sophisticated genetic screens have identified EphB4 mutations in some Vein of Galen Malformations and a small percentage of patients with capillary malformation-arteriovenous malformation syndrome^15–17^.

We hypothesized that there is a ratio of EphrinB2 to EphB4 that is critical for normal vasculogenesis and that this signaling ratio is abrogated in cerebral AVMs. In this study, we sought to understand the dynamic relationship between EphrinB2 and EphB4 in the normal and disease states to identify putative therapeutic targets and to investigate the potential utility of EphrinB2 as a quantifiable and therapy-responsive biomarker of pediatric AVM.

## Materials and methods

### Cell culture

Human brain microvascular endothelial cells (HBMVECs) were supplied by Cell Systems (Kirkland, WA). AVM-derived endothelial cells (AVMECs) were cultured primarily according to an Institutional Review Board-approved protocol. Fresh AVM tissue was dissociated in collagenase/dispase (Roche, Indianapolis, IN) at 37 °C, pelleted at 1200 RPM × 5 min, reconstituted in EGM2 media with 5% serum (Lonza, Walkersville, MD) and plated on attachment factor-coated plates (GIBCO, Grand Island, NY). At near-confluence (~1 week of culture), sorting for CD31+ endothelial cells was performed using Invitrogen Dynabeads (Invitrogen, Carlsbad, CA) according to the manufacturer’s protocol^18^. Both the CD31− population and purified CD31+ cell populations were resuspended in growth media and plated. Under the microscope, CD31+ cells were visualized with magnetic beads still attached. Endothelial identity was confirmed by immunocytochemistry staining for CD31 (BD Pharmingen Cat. No. 555444, 1:200; BD Biosciences San Jose, CA).

### Immunofluorescence

AVMECs and HBMVECs were fixed in 4% formaldehyde on glass slides, permeabilized in 0.1% Triton X-100 and blocked in 10% goat serum. Cells were stained with CD31 (CD31 1:200, BD Biosciences San Jose, CA) or VE-cadherin (CD311:200, BD Biosciences San Jose, CA; VE-cadherin 1:200, Abcam, Cambridge, MA), and then they were incubated with an Alexa Fluor 594 goat anti-rabbit secondary antibody (Life Technologies, Grand Island, NY) and were counterstained with DAPI.

### Western blot analysis

Whole-cell protein extracts were prepared from cells at passage 8 that were lysed with cold RIPA buffer (Bio-Rad, Hercules, CA) that was supplemented with proteinase and phosphatase inhibitor (Thermo Scientific, Waltham, MA). Protein concentration was determined with a Bradford assay (Bio-Rad, Hercules, CA). Then, 20 µg of total protein was loaded onto a NuPage 4–12% Bis-Tris Gel (Thermo Scientific, Waltham, MA) with a Precision Plus Protein Kaleidoscope standard (Bio-Rad, Hercules, CA), which was followed by the transfer of proteins onto a nitrocellulose membrane (Bio-Rad, Hercules, CA). CD31 (BD Biosciences San Jose, CA), αSMA (Abcam ab5694; 1:1000; Cambridge, MA), EphrinB2 (Abcam, ab131536; 1:1000; Cambridge, MA) and EphB4 (Abcam, ab64820; 1:1000, Cambridge, MA) were detected and compared to GAPDH, which was the housekeeping protein control (EMD Millipore MAB374 1:5000; Billerica, MA).

### Reverse transcription and quantitative PCR

RNA was harvested from AVMECs (passage 6) and HBMVECs (passage 11) (RNeasy Mini Kit (Qiagen, Valencia, CA)). cDNA was synthesized (Superscript Vilo cDNA Synthesis Kit (Life Technologies, Grand Island, NY)) and amplified using gene-specific primers/probe sets for *GAPDH, EphrinB2* and *EphB4*. All PCRs were performed using Applied Biosystems Universal PCR Master Mix and Taqman Gene Expression Assays primer/probe sets (Thermo Fisher Cat. 4331182, Waltham, MA; Human Ephrin-B2 ID: Hs00187950_m1; Human Ephrin-B4 ID: Hs00174752_m1; Human GAPDH ID: Hs03929097_g1). Graphs represent the mean of duplicate experiments run in triplicate and normalized to GAPDH ± SE.

### Migration and invasion assays

Migration assays were performed using Costar 24-well Transwell plates (Corning, Tewskbury, MA). AVMECs (passages 6–10) and HBMVECs (passages 9–10) were plated at 100,000 cells/well in 100 μL of EGM2 media (Lonza, Walkersville, MD) with 0.1% serum in the upper chamber. In the lower chamber, either EGM2 with 0.1% serum (negative control), EGM2 full serum media (positive control), or EGM2 with 0.1% serum supplemented with EphrinB2-FC recombinant 250 ng/mL (R&D Cat. No. 7397-EB-050, Waltham, MA) or 100 nM EphB4 inhibitor^19^ (Sigma, NVP BHG 712, St. Louis, MO) were added to the lower chamber to act as a chemoattractant. After 24 h, cells remaining in the upper chamber were aspirated; cells in the bottom chamber were fixed, permeabilized, and stained using Diff-Quik Stain Set (Siemens, Malvern, PA) as previously published^20,21^. Invasion assays were performed in a similar fashion as above using Matrigel invasion chambers (Thermo Fisher Scientific, Waltham, MA). Experiments were performed in duplicate for each cell line, with a total of ten high-power fields analyzed per cell line. Representative images were captured at ×100, and the number of migrated (or invaded) cells was quantified using ImageJ software (National Institutes of Health, Bethesda, MD).

### Tube forming assay

Tube formation was assessed as previously published^21,22^. Both AVMECs and HBMVECs were grown to ~80% confluence and then were split into a 48-well dish coated with Matrigel Matrix Basement Membrane (Corning, Tewksbury, MA). Cells were resuspended in EGM2 media with 0.5% serum alone or with either EphrinB2-FC 250 ng/mL or NVPBHG712 100 nM, and then they were plated at a density of 1 × 10^5^ cells/well. After incubation at 37 °C for 18 h, the formation of tube-like structures was evaluated. Images of live cells were captured at ×100 magnification, and the number of completed junctions was quantified using ImageJ software (National Institutes of Health, Bethesda, MD).

### Patient population

Tissue and urine specimens were collected as previously published^21,23^ in accordance with protocols approved by the Boston Children’s Hospital IRB (IRB#10-417); informed consent was obtained. Only patients with magnetic resonance imaging (MRI) of the central nervous system were included. In total, 73 patients were included, 30 of whom had a diagnosis of AVM of which half had hemorrhaged (*n* = 15) and the other half were nonhemorrhaged (*n* = 15). The date of urine collection from hemorrhagic patients ranged from 5 days to 4 months post-hemorrhage. Control urine samples were collected from healthy age- and sex-matched control patients (*n* = 29), all of whom had CNS imaging to confirm the absence of any neoplastic or vascular disease in the brain or spine. Additional patient cohorts for comparative vascular analysis included pediatric patients with a diagnosis of moyamoya (*n* = 14).

### Clinical data review

The following clinical data were reviewed: AVM size, location, hemorrhage status, associated aneurysms, presence or absence of deep venous drainage as well as any additional comorbid disease or important clinical information. All angiographic imaging was reviewed, and AVM flow was graded by a pediatric neurointerventional radiologist (Orbach).

### Urine collection

As previously reported by our group and in accordance with an IRB-approved protocol, urine samples were collected preoperatively between 2006 and 2014^21,23–25^. No patient had evidence of other major physiologic stressors, such as stroke, recent surgery, chemotherapy or radiation, at the time of collection. Samples were collected, transported on ice and stored at −80 °C.

### Tissue collection

Tissue specimens were obtained from the Division of Neuropathology at Children’s Hospital Boston through the Dana Farber Cancer Institute-Integrated Tissue and Clinical Data Bank for Patients with Neurological Disorders and in accordance with an Institutional Review Board-approved protocol. For immunohistochemistry staining, representative slides of AVMs were prepared from paraffin-embedded tissue. Normal human brain arterial sections were obtained commercially (Genetex, Irvine, CA).

### Urinary and tissue biomarker analysis

#### Enzyme-linked immunosorbent assay (ELISA)

Biomarker profiling was performed on all patient urine samples in duplicate. Commercially available ELISA assays were procured to test EphrinB2 **(**US Biological Life Sciences, Cat No. 024841, Salem, MA). Assays were performed per company protocol after determining the urine protein concentration by Bradford assay, as previously reported^21,23–25^.

#### Immunohistochemistry

Representative, paraffin-embedded 10-μm sections of AVMs were stained as previously described using antibodies to EphrinB2 (1:100, Sigma HPA008999, St. Louis, MO)^23^. Staining was quantified using ImageJ software. For each patient, ten separate fields were reviewed at ×200 magnification, and the percentage of positive cells was calculated. The values from these ten fields were then averaged to obtain the overall percentage of cells demonstrating immunoreactivity for each patient.

#### Statistical analysis

Statistical analysis was performed with the help of a biostatistician (D.Z.). Power analysis for the biomarker study indicated that the sample sizes of 30 AVM, 29 control and 14 moyamoya patients provided 80% power to detect twofold differences in median levels of the urinary biomarkers using a nonparametric Mann−Whitney *U* test with a two-sided *α*-level of 0.05 (nQuery Advisor version 7.0, Statistical Solutions, Cork, Ireland). Box-and-whisker plots were used to represent median and interquartile ranges for each biomarker comparison^26^. Receiver operating characteristic (ROC) curve analysis was applied to assess the predictive accuracy of the biomarkers with area under the curve (AUC) and 95% confidence intervals (CI) as indices of diagnostic performance in differentiating between AVM and controls, as well as AVM and moyamoya^27^. Multivariable stepwise logistic regression was used to confirm that EphrinB2 was a predictive noninvasive urinary biomarker for differentiating between AVM patients and controls as well as between AVM and moyamoya patients independent of age and sex as covariates; a likelihood ratio test was used to assess significance^28^. Youden J-index in ROC analysis was used to identify the optimal cutoff values (pg/μg) for EphrinB2, and based on these cutoff values, sensitivity, specificity and accuracy were calculated^29^. Statistical analysis was conducted using IBM/SPSS software (version 21.0, IBM, Armonk, NY). Paired sample *t* test analyses were performed for the in vitro assays. Two-tailed values of *p* < 0.05 were considered statistically significant.

## Results

### AVM-derived endothelial cells (AVMECs) express CD31

**Fig. 1:**
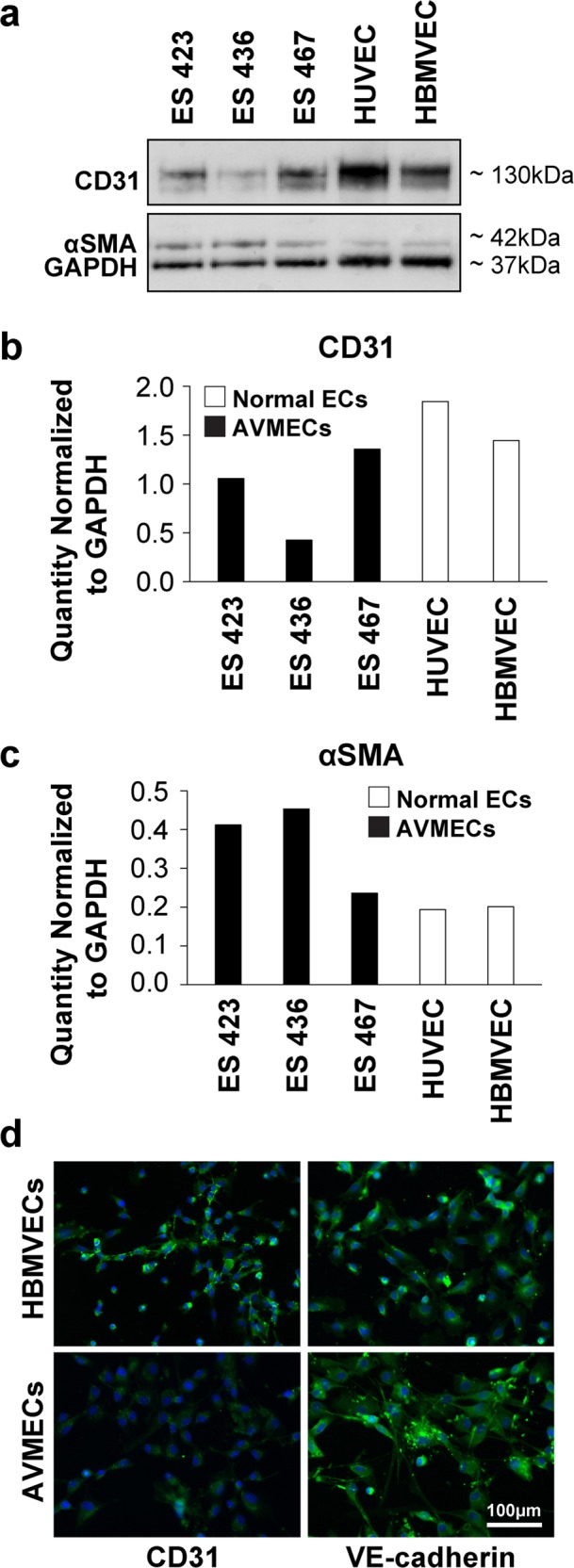
Characterization of patient-derived AVMECs. Primary endothelial cell lines were isolated from the human AVM tissue of three separate patients. The “purity” of primary patient-derived AVMECs was tested by Western blot (**a**) analysis using CD31 as an endothelial marker and αSMA as a mesenchymal marker. AVMECs express levels of CD31 (**b**) and αSMA (**c**) that are comparable with those of commercially available normal vascular endothelial cell lines. AVMEC endothelial identity was further confirmed by immunofluorescence that identified positive expression of endothelial lineage markers (CD31 and VE-cadherin). CD31 was also analyzed by Western blot and immunofluorescence, and VE-cadherin was analyzed by immunofluorescence (**d**) (CD31 and VE-cadherin = green, DAPI (nucleus) = blue). Original magnification ×200. Interestingly, VE-cadherin expression in AVMECs is not observed at the junctions, which is a pattern often noted in endothelial to mesenchymal transition.

AVMECs were isolated from primary AVM tissue based on a previously published method of CD31+ cell selection^18^ (Fig. 1). Endothelial lineage was confirmed in both AVMECs and HBMVEC controls by CD31+ immunocytochemistry (Fig. 1). Both CD31 and αSMA expression was further quantified by Western blot analysis to assess the level of mesenchymal impurity. The mesenchymal plasticity of AVMECs has been previously reported^30^ (Fig. 1). We found that the level of AVMEC mesenchymal impurity was actually comparable to that of commercially available endothelial cell populations, and it was similar to the HBMVEC control line. Interestingly, AVMEC VE-Cadherin staining localizes in a pattern often seen in endothelial to mesenchymal transition^31^. However, this may also be seen in endothelial barrier breakdown and inflammation^32^.

### The ratio of EphrinB2 to EphB4 is altered in pathologic AVMECs at both the mRNA and protein levels

**Fig. 2:**
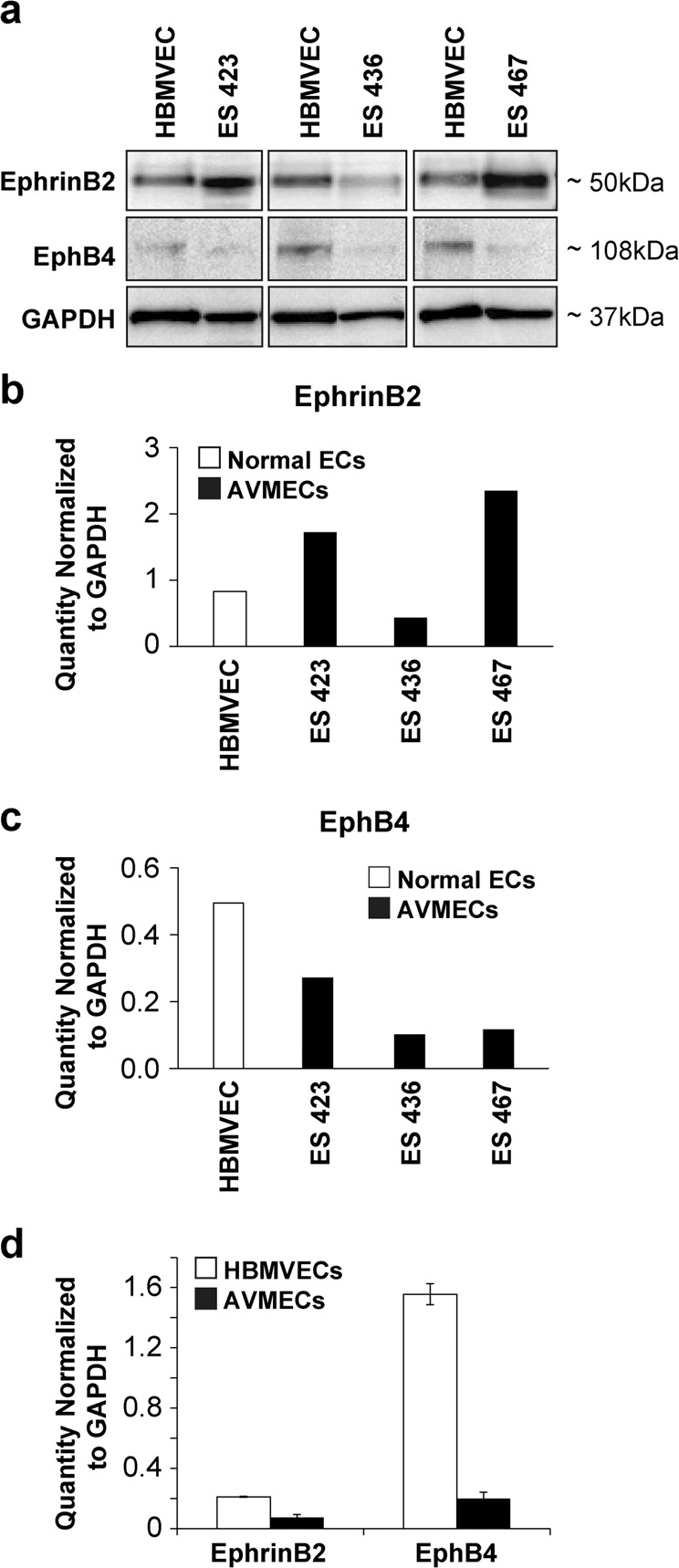
The ratio of EphrinB2 to EphB4 is altered in pathologic AVMECs at both the mRNA and protein levels. **a**–**d** Relative to control cells, the ratio of relative EphrinB2 to EphB4 is increased in AVMECs. **a** Western blot analysis demonstrated increased EphrinB2 and decreased EphB4 levels in AVMECs compared to those of normal HBMVEC controls. **b** On average, AVMECs express higher levels of EphrinB2, following normalization to GAPDH. **c** AVMECs express lower levels of EphB4, following normalization to GAPDH. **d** At the mRNA level, HBMVECs express higher levels of both EphrinB2 (threefold) and EphB4 (eightfold) at the mRNA level and a >3-fold higher ratio of EphB4 to EphrinB2 than what is observed in control cells.

At the mRNA level, AVMECs express *EphrinB2* at levels comparable to those of HBMVECs, but the levels of *EphB4* are diminished compared to those of the HBMVECs (Fig. 2). HBMVECs demonstrate a >3-fold higher ratio of receptor (EphB4) to ligand (EphrinB2). Comparison of AVMECs to HBMVECs at the protein level by Western blot demonstrates increased expression of EphrinB2 (~50 kDa) and decreased EphB4 expression (~108 kDa) across multiple AVMEC lines. The ratio of EphrinB2 to EphB4 is pathologically altered in the AVM endothelium.

### Compared to normal HBMVECs, AVMECs invade and migrate more and demonstrate impaired tube formation

**Fig. 3:**
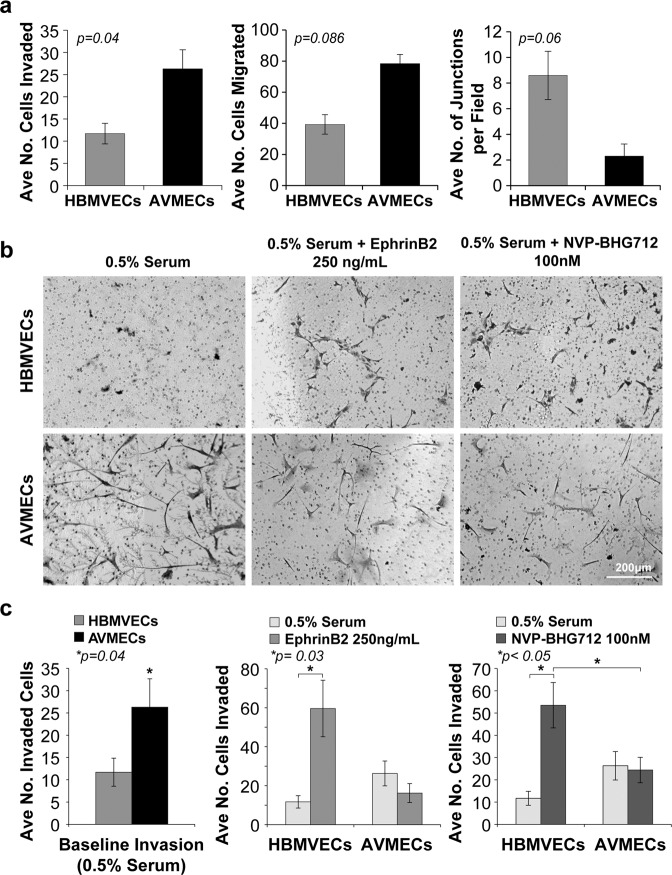
Angiogenic differences between control HBMVECs and AVMECs are demonstrated prior to treatment and are further augmented by altering the EphrinB2 to EphB4 ratio. **a** Baseline differences are observed between pathologic AVMECs and normal HBMVECs. AVMs had increased rates of invasion (*p* = 0.04) and migration (*p* = 0.086) and decreased tube formation (*p* = 0.06). Perturbations to the EphrinB2/EphB4 ratio (either increasing EphrinB2 or decreasing EphB4) can alter the phenotype of normal HBMVECs to mimic an exaggerated AVM response. **a**, **b** At baseline (0.5% serum), AVMEC invasion was at a rate that was nearly double that of HBMVECs (*p* = 0.04). **b**, **c** Stimulation with 250 ng/mL exogenous EphrinB2 increased HBMVEC invasion (*p = 0.03). **b**, **c** Inhibition of EphB4 with 100 nM NVP BHG increased HBMVEC invasion (*p < 0.05).

We next sought to understand the implications of the EphrinB2 to EphB4 ratio on angiogenesis in both AVMECs and HBMVECs (Fig. 3a). As a first step, we investigated basal differences between pathologic ECs and healthy controls by standard angiogenesis assays including migration, invasion and tube formation. Compared with normal HBMVECs, AVMECs exhibited higher rates of invasion (*p* = 0.04) and, though not statistically significant, a trend towards increased migration (*p* = 0.086) and decreased tube formation (*p* = 0.06). The healthy control HBMVECs demonstrated a greater number of completed junctions, polygon formation and early capillary-like structures, while the AVMECs appeared to have stagnate sprouting, fewer completed junctions and no polygon or capillary-like formation.

Of note, there is no universally agreed upon method for evaluating in vitro tube formation. Earliest methods focused on measuring tube length and absolute numbers of connected cells. More recent publications have focused on a combination of quantitative and qualitative measurements measuring branching/sprouting as well as assessing tube length and absolute connections. Here, healthy endothelial cells and AVMECs appear to peak at different points in the pathway to tube formation. Given this, angiogenesis was evaluated both by absolute connections and additionally by semi-quantitative evaluation of sprouting, connected cells, polygon formation, capillary-like structures and complex mesh^22^.

### Altering the EphrinB2 to EphB4 ratio increases HBMVEC invasion

To understand the role of altered EphrinB2 to EphB4 ratios in the AVMEC phenotype, we sought to induce AVM-like behavior in HBMVECs by increasing EphrinB2 levels or decreasing EphB4 levels (Fig. 3b, c). Of the tested parameters, baseline invasion was most significantly different between the two cell populations, so we focused first on investigating the role of EphrinB2/EphB4 signaling on invasive capacity. Before manipulation, AVMECs had a higher invasion capacity than HBMVECs. Upon stimulation with exogenous EphrinB2 (250 ng/mL), there was no significant difference in the AVMEC populations (*p* = 0.06); however, subjectively, there appeared to be decreased invasion. On the other hand, HBMVECs behaved similarly to AVMECs and exhibited invasion that was increased from the baseline (*p* = 0.03) and was greater than that of the AVMECs (*p* = 0.056). Similarly, inhibition of EphB4 with 100 nM NVP BHG 712 induced increased HBMVEC invasion (*p* = 0.02), which was also increased with respect to AVMECs (*p* = 0.047). Neither stimulation with EphrinB2 nor inhibition of EphB4 with NVP BHG 712 altered AVMEC invasion. Here, we show that by increasing the ratio of ligand to receptor either by increasing the ligand or by blocking the receptor, we can stimulate normal HBMVECs to behave like and phenotypically imitate AVMECs.

### Altering the EphrinB2 to EphB4 ratio results in increased tube formation in HBMVECs and increases the rate of disorganized AVMEC tube formation with redundant sprouting

**Fig. 4:**
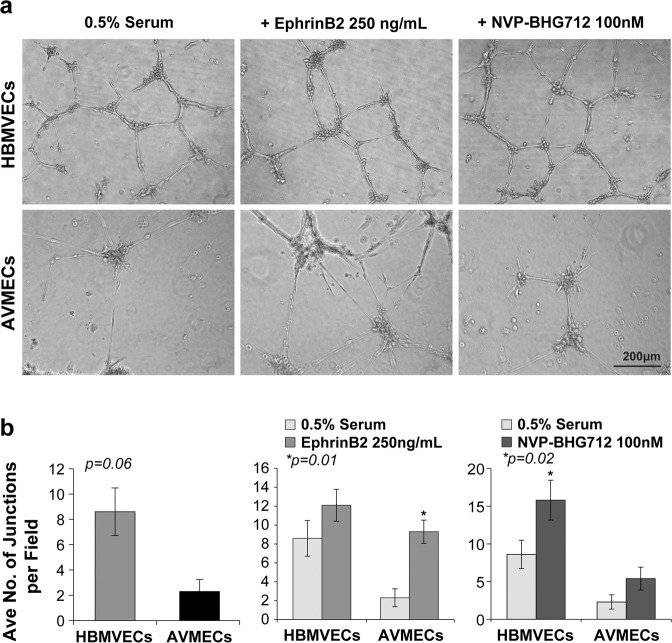
Altering the EphrinB2 to EphB4 ratio results in increased tube formation in HBMVECs and increases the rate of disorganized AVMEC tube formation with redundant sprouting. At baseline, AVMECs exhibit poor tube formation relative to that of controls. This can be seen qualitatively by incomplete tube formation and sprouting (**a**) as well as quantitatively by the number of completed junctions (**b**). Augmenting the EphrinB2/EphB4 ratio by stimulating with exogenous EphrinB2 increases the number of junctions (*p = 0.01) (**b**), although sprouting is redundant and disorganized compared to that of HBMVECs (**a**). Inhibition of EphB4 does not alter AVMEC tube formation (these cells are known to have low endogenous EphB4 expression); EphB4 inhibition subtly but consistently increases the total number of completed junctions in HBMVECs (*p = 0.02).

As noted above, tube formation was determined by quantifying the number of completed junctions with high-power microscopy; an additional qualitative assessment was performed to assess the degree of organization with respect to sprouting, connected cells, polygon formation, capillary-like structures and complex mesh (Fig. 4). Before treatment, AVMECs demonstrated predominant sprouting and a lack of completed junctions. AVMECs demonstrated an increased number of completed junctions (*p* = 0.01) in response to increased exogenous EphrinB2; however, the observed pattern remained simple, with redundant branching and sprouting and without demonstrable polygon or capillary-like formation. EphB4 inhibition had a minimal effect on AVMEC tube formation with respect to completed junctions, although there did appear to be a closer approximation to polygon formation than was observed in the untreated cells. Conversely, HBMVECs demonstrated a trend towards increased capillary-like tube formation and increased complexity in the presence of exogenous EphrinB2, and there was a statistically significant increase in tube formation in response to EphB4 inhibition (*p* = 0.02). Unlike what was observed in AVMECs, tube formation appeared organized in treated HBMVECs. This may reflect inherent differences between AVMECs and HBMVECs in addition to the Ephrin ratios that contribute to AVM pathology.

### Clinical investigation of EphrinB2 as a diagnostic marker

We next sought to understand whether increased levels of EphrinB2 would be clinically significant and useful as a diagnostic test. To study this, we collected patient samples as previously described and specifically assessed urinary EphrinB2.

#### Baseline characteristics of the patient population

A total of 73 patients were included in the study, of whom 30 had AVMs (Tables 1 and 2). There was no drop out, as the study focused on patients at the time of diagnosis. Longitudinal follow-up is ongoing. A total of 43 other patients were included in the study: 14 had moyamoya, and 29 were matched control patients. Demographics and clinical details are presented in Tables 1 and 2, respectively. Of the AVM patients, 15 were known to have had a prior hemorrhage as recently as 5 days prior to urine collection (2 patients were within 3 weeks of hemorrhage, and the remainder were collected >1 month post-hemorrhage). No patients were undergoing adjuvant treatment at the time of collection.

**Table 1:** Demographics and Ephrin-B2 levels of the study groups.

Variable	AVM	Moyamoya	Controls	P value
Gender (M/F)	15/15	2/12	10/19	0.088
Age, years	11 (8–13)	9 (3–16)	8 (4–12)	0.069
Ephrin-B2, pg/µg	35.0 (18.4–84.1)	0.0 (0.0–17.2)	11.7 (4.4–19.1)	<0.001*

Comparative demographics for all study patients revealed no statistically significant differences in age or sex. The table shows summarized univariate analysis highlighting AVM-specific biomarkers compared to controls and other CNS vascular diseases. By univariate analysis, urinary EphrinB2 showed statistically significant increases in patients with AVM versus matched controls as well as versus moyamoya. *p < 0.001. Biomarker data are summarized using medians (IQRs), and a nonparametric Mann−Whitney *U* test was used to compare each study group to AVM.

**Table 2:** Clinical characteristics of the included AVM patients.

Patient	Sex	Age	Nidus size	Location	Flow	Hemorrhage	Aneurysm	Deep venous drainage	Other	Urinary EphrinB2 (pg/µg)
1	M	12 y	5.2 mm × 7.55 mm × 7.49 mm	R subfrontal	Slow	N	Y	N	Pineal cyst	69.17047568
2	M	6 y	1.78 cm × 1.89 cm × 1.4 cm	R cerebellar	Slow	N	N	N		3.617056579
3	F	10 y	2.0 cm × 2.29 cm × 2.199	L frontal	Moderate	N	N	N		80.24224841
4	M	8 y	7 mm × 4 mm × 6 mm R-parietooccipital and 4 mm L-occipital	R occipitoparietal	Moderate	N	N	N	HHT- pulmonary AVM, R occipitoparietal, L occipital lobe AVMs; pineal cyst	21.40139505
5	M	17 y	11.8 mm × 10.4 mm × 14.4 mm	R frontal	Moderate	N	N	N	R occipital arachnoid cyst	607.41117
6	M	16 y	13.4 mm × 13.4 mm × 33.6 mm	R occipital	Fast	N	N	N		274.9936338
7	M	8 y	6.7 mm × 5.5 mm × 18.5 mm	R parietal	Slow	N	N	N	Pseudotumor	35.57083868
8	F	10 y	4.8 mm × 13.7 mm × 6 mm	R temporal	Slow	N	N	N		13.67116296
9	M	20 y	43 mm × 35 mm × 27 mm	R occipital	Fast	N	N	Y		19.79345955
10	M	6 y	Unknown	R cerebellar	Moderate	N	N	N	Recurrent AVM	26.68017562
11	F	16 y	6.8 cm × 3 cm × 4.8 cm	R parietooccipital	Fast	N	N	Y		9.668138035
12	M	12 y	2.4 cm × 1.89 cm × 2.5 cm	R posterior temporo-occipital	Fast	N	N	N	Fragile X	126.9329364
13	F	3.5 y	1.5 cm × 1.1 cm × 1.5 cm	L frontotemporal	Fast	N	Y	N	Onyx embolization prior to urine collection	65.03312118
14	F	9 y	2.7 cm × 3.3 cm × 3 cm	R occipital	Fast	N	N	N		153.1746032
15	M	11 y	1 cm × 6 mm × 7 mm	R parietooccipital	Moderate	Y	Y	Y		28.64676121
16	M	7 y	2.6 cm × 2.2 cm × 1.8 cm	R occipital	Slow	Y	N	N		30.125
17	F	13 y	5.7 mm × 4 mm x 5 mm	L occipital	Slow	Y	N	N	DVA	72.08640107
18	F	6 y	2 mm × 1 mm × 27 mm	L cerebellar	Fast	Y	N	Y		52.14877029
19	F	12 y	9.9 mm × 6.9 mm × 9.2 mm	R intraventricular	Slow	Y	N	Y		2.890728708
20	F	8 y	9 mm × 7 mm × 4.8 mm	L occipital	Slow	Y	N	N		27.3550884
21	F	12 y	4.6 mm × 6.7 mm × 4.2 mm	L parietal	Moderate	Y	N	N		95.65313735
22	F	15 y	18.5 mm × 15.2 mm × 11.1 mm	L occipital	Moderate	Y	N	N	Arachnoid cyst, melanoma of lip	27.76888026
23	M	5 y	2.6 cm × 0.8 cm	R choroid plexus	Fast	Y	N	Y		34.41276728
24	F	15 y	Unknown	L occipital	Fast	Y	N	Y	Multiply recurrent AVMs	122.8041682
25	M	9 y	23.6 mm × 20.8 mm × 19.6 mm	L frontal	Slow	Y	N	Y		6.434498708
26	M	16 y	7.8 mm × 8.4 mm x 6 mm	L temporal	Moderate	Y	Y	N		521.7267687
27	F	8 y	2.0 cm × 1.6 cm × 3.4 cm	L temporal	Slow	Y	N	Y		7.356274413
28	F	8 y	1.4 cm × 1.5 cm × 1.4 cm	R cerebellar	Fast	Y	N	N		49.78739701
29	F	13 y	1.8 cm × 1.4 cm × 1.1 cm	R frontal	Moderate	N	N	N	Family history of autoimmune disorders, basilar stenosis at AICA	42.2308871
30	M	5 y	1.3 cm × 1.2 cm × 1.0 cm	R temporal	Moderate	Y	N	N		14.09318124

The following clinically relevant categories were reviewed for each patient included in this study: sex, age, AVM size, location, rate of flow, whether the AVM had hemorrhaged, presence of aneurysm, deep venous drainage, and any associated conditions. Patients with the highest urinary EphrinB2 levels were noted to have high-flow lesions and/or had associated aneurysm or other pathology. Patients with urinary levels that fell below the cutoff (<25 pg/µg) had either slow-flow lesions or lesions with deep venous drainage.

Univariate comparisons between AVM patients and control subjects did not show relevant differences by age or sex (Table 1). No control subjects had documented history of tumors, vascular malformations or recent surgery (defined as within 3 months of specimen collection). No AVM, moyamoya, or control patient was critically ill or receiving any adjuvant therapies at the time of sample collection.

#### Measurement of urinary EphrinB2 demonstrates clinical relevance as a biomarker

Levels of EphrinB2 in urine were quantified using a commercially available ELISA assay. The results were normalized to overall urinary protein concentration, and levels from AVM patients were compared to levels from control patients. Subsequent analyses were performed to compare biomarker levels from AVM patients with biomarker levels in patients with other pediatric cerebrovascular disease (moyamoya). Statistically significant differences in biomarker expression were identified by multivariate analysis. (Table 1).

#### EphrinB2 distinguishes AVMs from controls

**Fig. 5:**
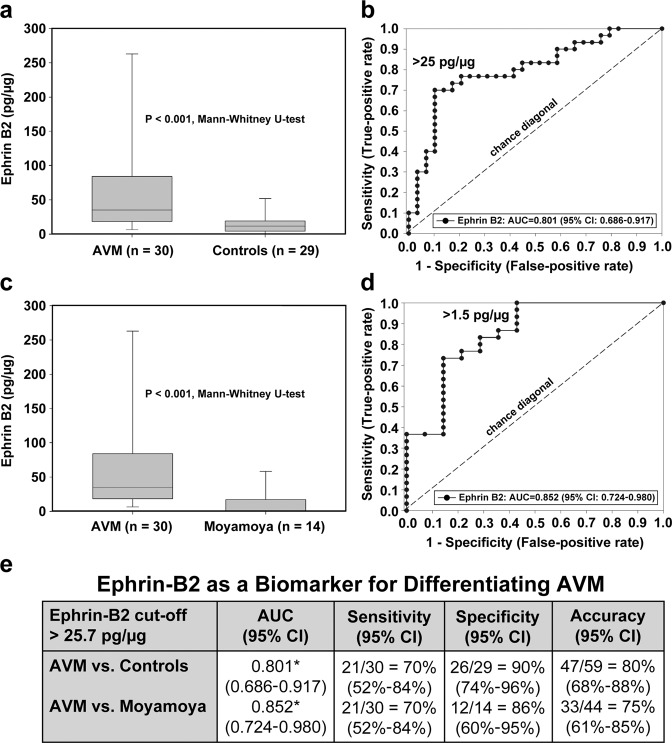
EphrinB2 as a biomarker of pediatric AVM: Urinary EphrinB2 distinguishes AVM patients from controls and from patients with other cerebrovascular disease. **a** Urinary levels of EphrinB2 are capable of distinguishing patients with AVM from healthy matched controls (*p* < 0.001). The median levels of urinary EphrinB2 were 35.0 pg/µg (IQR 18.4–84.1) for AVM patients and 11.7 pg/µg (IQR 4.4–19.1) for controls. **b** Regression modeling demonstrated that urinary biomarkers can predict the presence of AVM with high specificity and sensitivity. A urinary EphrinB2 value > 25 pg/μg was seven times more likely to be present in patients with AVM (AUC 0.801, 95% CI) than it was in controls, and this result was independent of age and sex. **c** Example of the ability of urinary biomarkers to discriminate between different types of CNS vascular disease. By univariate analysis, levels of urinary EphrinB2 could distinguish between patients with AVM and other cerebrovascular disease (moyamoya) (*p* < 0.001). The median levels of urinary EphrinB2 were 35.0 pg/µg (IQR 18.4–84.1) for AVM patients and 0.0 pg/µg (0.0–17.2) for moyamoya patients. **d** Results of the regression modeling showed that a urinary EphrinB2 value > 25 pg/μg was five times more likely to indicate AVM presence (AUC 0.852, 95% CI). **e** ROC analysis of EphrinB2 showed excellent discrimination when used as an independent marker for AVM versus control with 80% accuracy and AVM versus moyamoya with 75% accuracy.

Multiple stepwise logistic regression analysis revealed that, independent of age and sex, urinary EphrinB2 was able to distinguish between AVM patients and controls (Fig. 5). Median levels of urinary EphrinB2 were 35.0 pg/µg (IQR 18.4–84.1) for AVM patients and 11.7 pg/µg (IQR 4.4–19.1) for controls (Fig. 5a).

Optimal cutoff point for EphrinB2—determined by ROC analysis and regression modeling—demonstrated that a urinary EphrinB2 value of >25 pg/μg has a sevenfold increased likelihood of AVM presence when compared to controls, and this was independent of age and sex (Fig. 5b). The AUC for EphrinB2 comparing AVM versus controls was 0.801 (95% CI, 0.686–0.917), with a sensitivity of 70%, a specificity of 90%, and an overall accuracy of 80% (Fig. 5e).

#### EphrinB2 distinguishes AVM from other cerebrovascular diseases

To establish that EphrinB2 is not just a ubiquitous marker of neurovascular pathology, we demonstrated that we could distinguish between AVM and ischemic vasculopathy (moyamoya) (Fig. 5). Median levels of urinary EphrinB2 were 35.0 pg/µg (IQR 18.4–84.1) for AVM and 0.0 pg/µg (IQR 0.0–17.2) for moyamoya patients (Fig. 5c). EphrinB2 is elevated in the urine of AVM patients compared to that of vascular (moyamoya) controls, with an AUC of 0.852 (0.724–0.908), a sensitivity of 70%, a specificity of 86%, and an overall accuracy of 75% (Fig. 5e). Using the same cutoff (>25 pg/µg), EphrinB2 was fivefold more likely to detect AVM presence than it was to detect moyamoya.

#### AVM tissue exhibits elevated levels of EphrinB2 expression

**Fig. 6:**
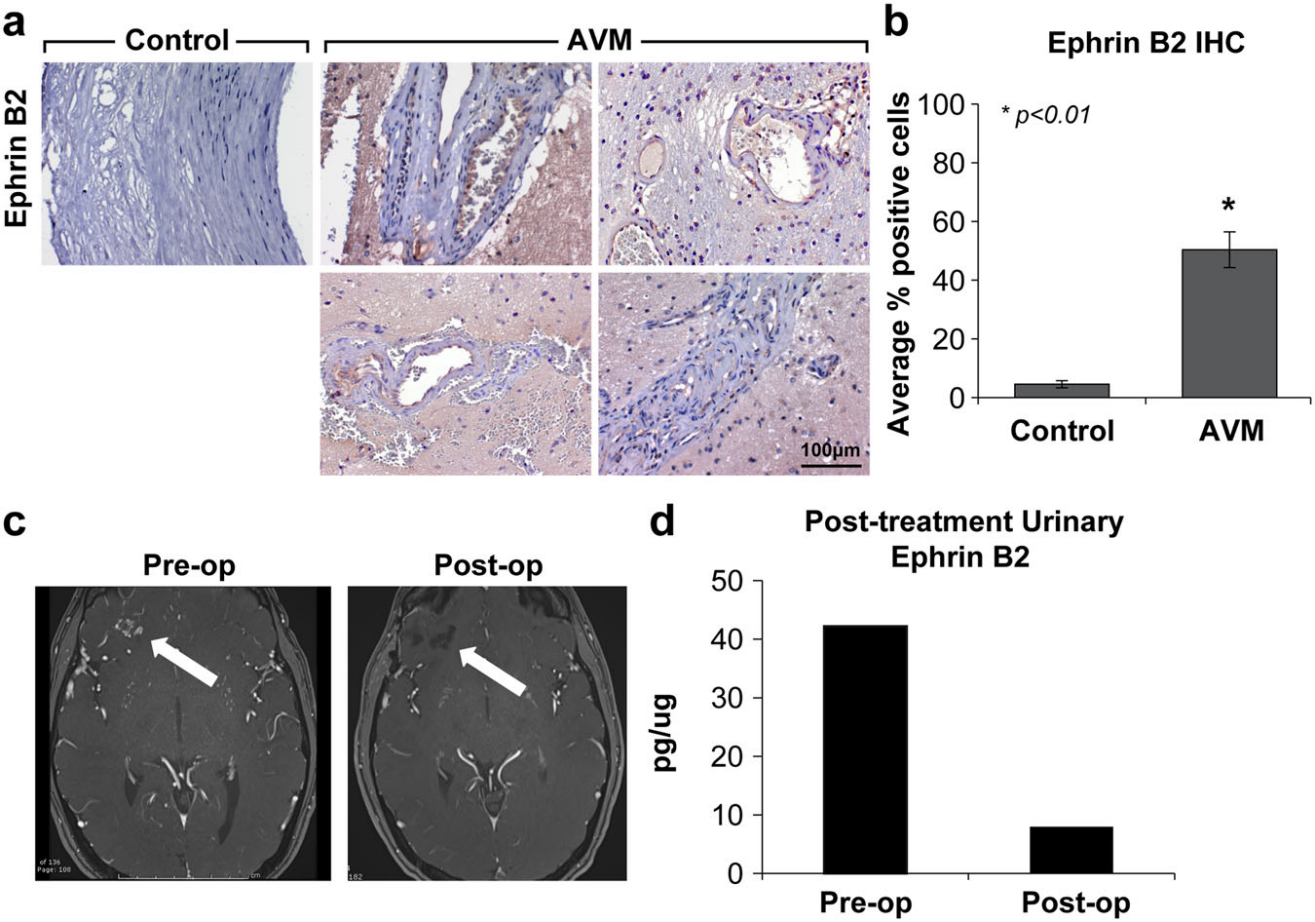
Strong EphrinB2 staining in AVM tissue and normalizing levels of urinary EphrinB2 post-resection suggest AVM tissue as the biomarker source. **a** Representative photomicrographs show four different AVMs as confirmed by neuropathology. Immunohistochemical analysis identifies AVM as a putative source of urinary biomarker levels. EphrinB2, which localizes primarily to the arterial endothelium with scattered perinidal expression, was detected in AVM tissue at significantly higher rates than in commercially available normal arteries. Diaminobenzidine and hematoxylin counterstain, original magnification ×200. **b** There was a tenfold increase in the number of cells with identifiable EphrinB2 staining over a normal artery control (*p < 0.01). **c** Index patient for whom pre- and post-treatment analysis could be performed: preoperative MRI shows a small right frontal AVM, and postoperative MRI demonstrates a gross total resection. **d** At the time of diagnosis, urinary levels of EphrinB2 (>40 pg/µg) exceeded the established cutoff. Following resection, urinary EphrinB2 normalized to below the diagnostic cutoff.

Urinary biomarker levels were correlated with AVM tissue expression, as determined by immunohistochemical (IHC) analysis (Fig. 6). Individual specimens with a diagnosis of AVM (*n* = 4) were obtained and subjected to IHC. EphrinB2 was detected in AVM tissue at higher rates than it was in normal artery control (*n* = 2). Staining for EphrinB2 in AVMs showed a tenfold increase over the normal control (*p* < 0.01). A heterogeneous population of cells, including nidal endothelial cells and perinidal cells, were noted to express varying levels of EphrinB2 (Fig. 6a, b). EphrinB2 has previously been shown to be expressed in the endothelium, smooth muscle cells, and pericytes during mouse development^33,34^.

#### EphrinB2 is a potential treatment-responsive marker

As proof of concept, in one patient for whom pre- and postoperative urine was available, we investigated whether EphrinB2 levels normalized subsequent to treatment (Fig. 6). Following gross total resection, EphrinB2 decreased ~8-fold and normalized to below the cutoff value (from 42.2 to 7.9 pg/µg) (Fig. 6c, d). Further longitudinal studies are ongoing.

### Correlation with clinical findings

The most important angiographic correlations with urinary EphrinB2 levels were rate of flow and presence of deep venous drainage (Table 2). Of the patients with the highest levels of EphrinB2 (>100 pg/µg), four of six had fast flow (i.e., angiographic opacification of draining veins within 500 ms of opacification of the arterial pedicles) and two of six had moderate flow lesions (i.e., opacification of draining veins within 1000 ms). Of the two with moderate flow, one patient was noted to have an aneurysm at the time of hemorrhage, and the other had a large AVM and an associated arachnoid cyst. Of the nine patients with urinary levels of EphrinB2 that were below the established cutoff (<25 pg/µg), six had slow-flow lesions (i.e., opacification of draining veins at > 1000 ms), five of nine had deep venous drainage, and two of nine lesions with below threshold levels of EphrinB2 were in fact fast flow lesions *with* deep venous drainage. We hypothesize that the role of deep venous drainage is an important consideration for the utility of EphrinB2 as an AVM biomarker. Most consistent with the aforementioned findings, the lesion in the patient for whom urinary EphrinB2 levels were lowest, at 2.89 pg/µg, had *both* slow flow and evidence of deep venous drainage. The last patient below the cutoff value had a medium flow lesion, no deep venous drainage, and importantly had hereditary hemorrhagic telangiectasia—a pathology distinct from the other isolated AVMs included in this study.

## Discussion

Cerebral AVMs are devastating, and there is a clear imperative for translational research to advance current standards of diagnosis and treatment, particularly in the pediatric population. The research described here increases the understanding of the role of AGF signaling in AVM pathology, establishes the importance of a specific EphrinB2:EphB4 ratio in vasculogenesis, and reveals a relative increase in EphrinB2 in AVMs as a result of imbalances in the Ephrin signaling ratio. Our findings reveal that the specific balance of EphrinB2:EphB4 ratios in endothelial cell signaling is an important regulator of key pathophysiological mechanisms in AVM biology. The novel insights reported in this research have immediate clinical application in biomarker development and highlight future innovative targets for AVM therapeutics that have implications for adult AVMs as well.

We first characterized our AVMECs and demonstrated that at baseline they have increased migration and invasion with impaired tube formation as compared to normal controls. This is consistent with prior characterization of AVMECs in the literature.^35,36^. When we compared our AVMECs with HBMVECs in terms of endogenous expression of EphrinB2 and EphB4, we found that although both cell lines expressed EphrinB2 and EphB4 at both the mRNA and protein levels, there was a marked increase in the EphrinB2 to EphB4 ratio in the AVMECs compared to the normal controls, which was most apparent at the protein level (Fig. 2). We have considered that the ratio of EphrinB2:EphB4 present in our AVMECs could be influenced by their arterial vs. venous nature; specifically, we considered whether or not the endothelial cells have been derived from AVM “veins” or AVM “arteries”. Ultimately, these cell lines represent a heterogeneous population, which ultimately recapitulates the endothelial subpopulations in AVMs. Grossly, there are no means by which we could accurately estimate what % of the specimen is an arterialized vein or what is a true artery vs. vein.

We evaluated the significance of this abrogated ratio in vitro. Of note, we modified the concentration of available protein to accomplish this instead of performing gene overexpression or silencing experiments. Soluble proteins that can target cell surface receptors are more amenable to the existing patient care framework, which includes microvascular access via direct angiography, and we therefore focused our research efforts with this in mind.

First, we increased the relative ratio of EphrinB2 to EphB4 in normal brain vascular endothelial cells to try to induce AVM-like behavior. By either increasing EphrinB2 or blocking EphB4 forward signaling, we observed invasion in these normal cell lines that increased to rates comparable to those of the AVM cells (Fig. 3). We further observed that an artificially increased EphrinB2:EphB4 ratio led to increased tube formation in the normal healthy cells, but unlike AVMECs, the pattern was better organized and exhibited polygons and capillary-like formation. The maintenance of organization in HBMVEC tube formation suggests that, as expected, there are additional factors contributing to AVMEC pathology (Fig. 4). In total, these data indicate that an increased EphrinB2:EphB4 ratio can promote AVM-like pathology in normal endothelial cells.

We then investigated the effect of further alterations on the EphrinB2:EphB4 ratio in AVM cell lines. We discovered that additional increases in the EphrinB2:EphB4 ratio by EphrinB2 stimulation in AVMECs resulted in a more pronounced AVM phenotype, as characterized by an increased number of junctions with redundant branch points, but there was no more complex polygon or capillary-like formation observed. Interestingly, EphB4 blockade did not increase the number of completed junctions, though qualitatively resembled a closer approximation to incomplete polygon formation. We attributed the more subtle impact of EphB4 inhibition in AVMECs to the previously demonstrated profound reduction in the endogenous expression of EphB4 in AVMECs compared to that of non-AVM ECs. These data further support the hypothesis that EphrinB2 and possibly reverse signaling imbalance contributes to pathologic angiogenesis in both control and AVM cell lines and that there may be a spectrum of responses dependent on the degree of imbalance. Ultimately, there are likely primary genetic drivers of cerebral AVM pathology that can be directly influenced by external signaling, such as EphrinB2:EphB4.

Given that the results of our in vitro studies demonstrated increased EphrinB2 ligand levels with subnormal levels of EphB4 receptor, we expected a measurable increase in EphrinB2 in our AVM patients. This hypothesis was validated when we measured urinary EphrinB2 levels in AVM patients and compared them to matched healthy controls. We identified a statistically significant increase in urinary EphrinB2 levels in AVM patients compared to control and other disease cohorts (*p* < 0.001) (Fig. 5). Importantly, urinary biomarker levels corresponded directly with primary AVM tissue expression, linking circulating biomarkers to source tissue (Fig. 6). Further substantiating this link, we presented evidence that resection of an AVM results in a concomitant reduction in urinary EphrinB2 levels, supporting the premise that the AVM is the source of this urinary marker (Fig. 6). Finally, we demonstrated that EphrinB2 may represent a specific biomarker “fingerprint” for AVM, as levels are not elevated in other pathological states, even other central nervous system vascular disorders, such as moyamoya (Fig. 5). These findings represent important, clinical, proof-of-principle data that urinary EphrinB2 merits further investigation as a specific, reliable, treatment-responsive biomarker.

To further understand the clinical utility of these data, we cross-referenced our findings against an important clinical feature of cerebral AVM which is flow. We hypothesized that AVM flow patterns influenced EphrinB2 levels. AVMs were angiographically stratified by flow (high-flow and low-flow), size and drainage patterns (deep and superficial) by a board-certified neuroradiologist. When correlated with urinary EphrinB2 levels, we found that high-flow lesions had higher levels of urinary EphrinB2, independent of size. In contrast, slow-flow lesions or AVMs with deep venous drainage had lower levels of EphrinB2.

One explanation for this finding is that EphrinB2 is expressed as a cell surface protein, and turbulence in high-flow AVMs may lead to increased disruption of the endothelium and possible release of EphrinB2. Independent of nidus size, fast flow directly correlated with higher levels of urinary EphrinB2 > 100 pg/µg. Conversely, slow-flow AVMs had urinary EphrinB2 levels below 35 pg/µg. Deep venous drainage also correlated with low urinary EphrinB2 levels. As venous vasculature has been reported to have higher expression of the receptor EphB4, one potential explanation for lower ligand levels in AVMs with deep venous drainage could be proximity to a greater surface area of receptor to absorb AVM-generated ligand. This speculative hypothesis needs additional investigation; however, regardless what the underlying mechanism is, these data are clinically relevant.

As with many clinical studies, there are limitations to what we can extrapolate from these data given the sample size and what we cannot control with respect to in vivo variables. While this work requires further validation with larger populations, we achieved statistical significance and provided substantial proof-of-principle data to encourage further investigation. A second issue that is true of any biomarker study of isolates is that we cannot control for in vivo variables, including inflammatory cells and other secreted cytokines, chemokines, and growth factors. In this vein, we recognize the heterogeneity of our AVMs, including both hemorrhage and nonhemorrhage patients. Ideally, subsets of each group could be analyzed independently; however, when we stratified the data based on prior hemorrhage, there was no significant difference between groups, suggesting that the underlying premise that EphrinB2 is a product of AVM cells (and not a byproduct of hemorrhage or trauma) is valid.

In the future, further in vitro and in vivo validation will be important, given the potential clinical relevance of these findings. Our data provide a diagnostic biomarker and as a potential therapeutic target. We identified a relationship between EphrinB2:EphB4 expression in AVMs and correlated EphrinB2 overexpression with key mechanistic drivers of pediatric AVM pathology in both in vitro and clinical studies. Further validation is warranted, as these results may have significance in the development of noninvasive biomarkers and novel Ephrin-derived small molecule therapeutics to modify the disease course of this devastating disease.
